# Femtosecond Laser Stealth Slicing of 4H-SiC Wafers with Static Aspheric Aberration Correction

**DOI:** 10.3390/ma19071292

**Published:** 2026-03-24

**Authors:** Tingkai Yang, Rong Wu, Xiangji Guo, Tao Chen, Ming Ming

**Affiliations:** 1 School of Physics and Optoelectronic Engineering, Hangzhou Institute for Advanced Study, UCAS, Hangzhou 310024, China; yangtingkaiw@ucas.ac.cn (T.Y.); guoxiangji@ucas.ac.cn (X.G.); 2 School of Applied Chemistry and Engineering, University of Science and Technology of China, Hefei 230026, China; wurong@ciac.ac.cn; 3 Changchun Institute of Optics, Fine Mechanics and Physics, Chinese Academy of Sciences, Changchun 130033, China; chent@ciomp.ac.cn

**Keywords:** 4H-SiC, femtosecond laser, aberration correction, stealth slicing, modified layer

## Abstract

Silicon carbide (SiC), owing to its excellent physical and chemical properties, has emerged as a leading third-generation semiconductor material. Conventional diamond wire cutting faces challenges in producing ultra-large, ultra-thin wafers. In contrast, the femtosecond laser has attracted significant attention in recent years due to its low kerf loss and high slicing speed. However, during femtosecond laser stealth slicing, spherical aberration induced by the refractive index mismatch between air and the SiC crystal severely degrades the slicing quality. Based on the analysis and calculation of wavefront aberration at a specific focal depth of 175 μm, we designed and implemented a static aberration correction method to reduce the thickness of the modified layer and improve the slicing quality. This method effectively mitigates focus elongation caused by refractive index mismatch, thereby reducing the modified layer thickness and the tensile stress required for wafer separation, while improving the surface quality of the separated wafers. Furthermore, this method eliminates the need for active optical components in aberration correction, simplifying the system and avoiding errors associated with the limited response speed of active optics. The technique demonstrates potential for practical application in industrial wafer slicing.

## 1. Introduction

Silicon carbide (SiC), owing to its wide bandgap, high thermal conductivity, high carrier saturation velocity, and excellent breakdown field strength, has emerged as one of the most promising third-generation semiconductor materials [1,2]. These properties make it well-suited for high-temperature, high-frequency, and harsh-environment applications in advanced power electronic devices [3,4,5]. More than 70 polytypes of SiC crystals have been identified, distinguished by their stacking sequences [6]. Among them, 4H-SiC exhibits superior crystal quality, higher Mohs hardness (9.3–9.5), lower drift layer resistance, and better thermal stability compared to other polytypes [7]. However, its exceptional physical and chemical properties also pose significant challenges for conventional mechanical wafer slicing. Currently, the most widely used method for SiC wafer separation is wire sawing using diamond-coated wires. This contact-based slicing technique suffers from several drawbacks, including excessive debris generation, high residual stress, and poor surface roughness [8,9]. In addition, the low cutting speed, poor processing efficiency, and low yield associated with diamond wire sawing, combined with a relatively large kerf loss of approximately 250 μm, significantly increase the overall manufacturing cost of SiC wafers [10].

In recent years, the femtosecond laser has emerged as a powerful tool for next-generation precision fabrication due to its exceptional focusing ability and extremely high peak power [11,12,13]. Femtosecond laser stealth slicing is a technique that focuses pulsed laser beams inside the crystal to induce internal modification, thereby enabling wafer separation with the aid of a laser-modified layer. Its non-contact nature avoids tool wear and mechanical stress effects [14,15]. Compared with long-pulse or continuous-wave lasers, ultrafast laser interacts with materials over extremely short timescales, effectively preventing heat accumulation and associated thermal stress damage. Therefore, ultrafast laser-based slicing can significantly reduce the modified layer thickness and improve the separation surface quality [16]. In femtosecond laser-based SiC wafer separation, various parameters directly influence the final processing quality, including pulse laser parameters (repetition rate, laser fluence, scanning speed), numerical aperture (NA) of the objective, and spot size [17,18]. In recent years, researchers have extensively investigated key parameters influencing the laser-based separation of SiC wafers. Eunho Kim et al. successfully achieved stealth slicing of SiC wafers using a dual-pulse ultrafast laser configuration. They further demonstrated that the dual-pulse sequence provided superior slicing performance compared to the single-pulse counterpart, yielding significantly narrower kerf loss than that obtained by conventional wire sawing [10]. Leger et al. conducted preliminary studies on the effect of numerical aperture by comparing material responses under single-pulse and burst modes, and highlighted the critical role of nonlinear propagation in ultrashort pulse interactions with materials [19]. Wenhao Geng et al. first used a femtosecond laser to generate a modified layer inside 4H-SiC. This modified layer mainly consists of amorphous silicon and amorphous carbon, with a bandgap of approximately 0.4 eV. Subsequently, this bandgap difference was utilized to achieve bandgap-selective corrosion during the photoelectrochemical etching process. The photogenerated holes preferentially oxidize and etch the laser-induced modified layer, while the upper and lower undamaged 4H-SiC regions remain intact, thereby enabling selective separation and slicing of the wafer along the modified layer [20].

During wafer separation, the collimated femtosecond laser is focused by an objective lens and enters the SiC wafer from the air at an oblique angle. The refractive index mismatch between air and the SiC crystal induces significant spherical aberration. According to Snell’s law [21], this aberration leads to degradation of the objective’s point spread function (PSF), focal shift, and elongation of the focal spot. Such distortions not only reduce the lateral machining resolution but also make it difficult to control the thickness of the modified layer. Fundamentally, this aberration arises from phase discontinuities in the wavefront during cross-media propagation, and its precise compensation remains a critical challenge in ultra-precision machining of wide-bandgap semiconductors.

To address the accuracy loss caused by aberration, researchers have commonly adopted active optical components integrated at the conjugate pupil plane of the objective lens. Among them, liquid crystal spatial light modulators (LC-SLMs) [22] and deformable mirrors (DMs) [23,24] have emerged as mainstream solutions. These dynamic wavefront shaping technologies offer real-time compensation for phase distortions induced by abrupt refractive index changes at material interfaces. In 2024, Wang et al. employed a Shack-Hartmann wavefront sensor to measure the aberrations carried by the supercontinuum emitted from plasma at various depths within the glass. A spatial light modulator (SLM) was then used to compensate for the aberrations based on both the measured data and theoretical calculations [25]. The results showed that the focal spot of the femtosecond laser achieved an aspect ratio of approximately 2.82–2.91 after aberration correction, significantly improving the focusing performance. However, implementing active aberration correction in femtosecond laser processing systems still remains technically demanding. Active wavefront correction systems require complex integration of wavefront sensors, real-time controllers, drive electronics, optical components, and algorithmic dependencies. In addition, various static methods for aberration correction have been extensively employed in recent years. In 2017, Margaux Chanal et al. proposed a solid-immersion focusing strategy, in which a specially tailored surface curvature is introduced on the sample to enable three-dimensional femtosecond-laser inscription in high-index, strongly nonlinear materials such as silicon [26]. In this experiment, multiple objectives with different numerical apertures (NAs) equipped with correction collars were employed to compensate for the spherical aberration induced by the silicon thickness. Although correction-collar objectives afford a degree of compensation for spherical aberrations caused by material thickness or refractive-index gradients, the underlying corrective action remains essentially a geometrical-aberration tuning. Without the solid-immersion structure, nonlinear effects—principally two-photon and free-carrier absorption—prematurely deplete energy near the surface, preventing the formation of a bulk focus with sufficient laser fluence. Moreover, under high-power femtosecond irradiation, the correction collar is susceptible to thermal drift and mechanical instability, thereby compromising long-duration operation.

To eliminate the spherical aberration caused by the refractive index mismatch, a static aberration correction method based on an aspheric lens was designed and introduced in this study. By pre-modulating the reference wavefront of the processing beam, the proposed method effectively compensates for aberrations induced by refractive index discontinuities, thereby improving focusing quality and optimizing the energy distribution of the beam. Experimental results confirm that this approach significantly reduces the tensile stress required for wafer separation, while simultaneously decreasing the modified layer thickness and surface roughness of the separated interface. The objective of this study is to achieve a modified layer thickness of less than 15 μm at a focusing depth of 175 μm, while ensuring that the surface roughness (Sa) after separation is below 5 μm. The results demonstrate that high-quality processing of large-area ultrathin wafers can be achieved without the need for active optical components, highlighting the method’s strong potential for practical engineering applications.

## 2. Experimental Setup and Method

### 2.1. Experimental Setup

**Figure 1:**
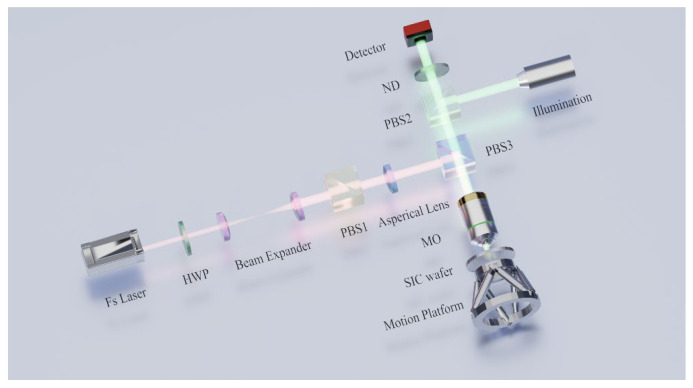
Schematic diagram of the femtosecond laser processing system for 4H-SiC wafer slicing.

As shown in Figure 1, the experimental setup employed in this study is illustrated. The femtosecond laser used in this study is the Pharos series from Light Conversion (Lithuania). Its parameters are as follows: average power P = 20 W, pulse duration 256 fs, central wavelength λ = 1030 nm, adjustable repetition frequency ranging from 1 Hz to 1 MHz, and a TEM00 mode with M^2^ < 1.2. At the fundamental repetition of 100 kHz, the maximum single-pulse energy is E = 200 µJ. The bandgap of 4H-SiC is approximately 3.26 eV, corresponding to a wavelength of about 387 nm. Therefore, at 1030 nm (~1.2 eV), the material remains transparent and single-photon absorption does not occur. However, femtosecond laser pulses at this wavelength can penetrate the crystal and initiate nonlinear interactions such as multiphoton absorption and avalanche ionization. Based on the bandgap of 4H-SiC and the single-photon energy of the laser, it can be deduced that the dominant nonlinear interaction inside the crystal is three-photon absorption. The wavelength of 1030 nm was chosen in this study to ensure internal energy deposition and effective modification without surface damage [18]. In addition, the 4H-SiC wafer used in this study exhibits a refractive index of n=2.60 and an extinction coefficient of $k\approx 10^{-5}$ at 1030 nm, which further confirms that linear absorption at this wavelength is negligible. After the initial ionization induced by the laser entering the crystal, avalanche ionization occurs under the strong electric field, rapidly generating a large number of free carriers. These carriers strongly couple and thermalize with the lattice on a picosecond timescale. In our experiments, the laser pulse duration was 256 fs, with an interval of 10 μs between adjacent pulses, while the electron–phonon relaxation time constant of 4H-SiC is approximately 0.63 ps [27]. This indicates that energy deposition is completed prior to thermal diffusion, enabling the heat-affected zone to be effectively confined within the micrometer scale and, consequently, allowing the damage region to be well controlled. The laser beam first passed through a half-wave plate (HWP) to adjust its linear polarization. After beam expansion, it was directed to a polarizing beam splitter (PBS) to improve the polarization extinction ratio of the pulses. Then, it is directed into a static aberration correction system to compensate for wavefront distortions. Finally, the beam is focused into the interior of the wafer using a 50× objective lens (Mitutoyo, Japan, M Plan Apo NIR × 50, NA = 0.42). To ensure normal incidence of the focused beam into the wafer, the wafer was mounted on a six-degree-of-freedom motion stage (PI, German, H-811.I2), which was secured to a self-developed two-axis motion stage. To observe the changes around the processing site during machining, a coaxial observation system was integrated.

**Figure 2:**
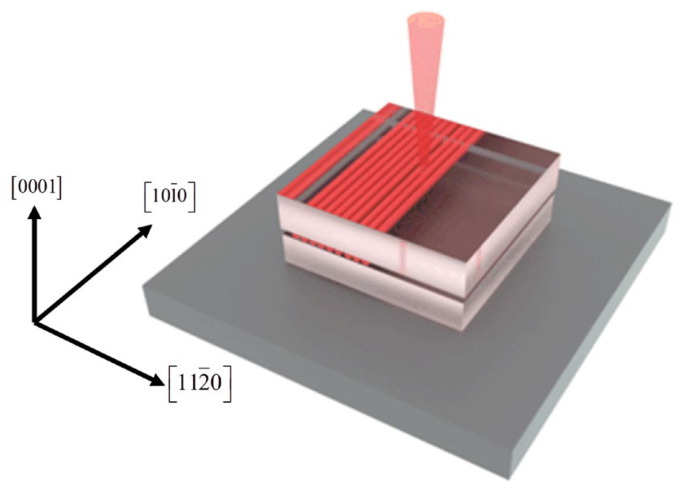
Schematic diagram of femtosecond laser scanning inside the crystal.

Femtosecond-laser processing parameters directly affect the processing outcome. As shown in Figure 2, this is a schematic diagram of femtosecond-laser scanning inside the SiC crystal. In 2023, Yang et al. polished reaction-bonded silicon carbide (RB-SiC) by a femtosecond laser and systematically analyzed the influence of laser parameters on the polishing results [28]. They identified the equivalent pulse number (EPN), laser fluence, and scanning interval as influential factors. The EPN is defined as the number of laser pulses incident on a single point of the sample during scanning and can be expressed as:

(1) $$N=\frac{{2\omega }_{0}}{v}f$$

Here, $\omega_{0}$ represents the radius of the focused spot, *v* is the scanning speed, and *f* is the repetition rate. These parameters jointly regulate the pulse-overlap statistics and the extent of energy deposition per unit area, thereby markedly influencing the morphology, continuity, and fracture behavior of the laser-induced modified layer.

Moreover, these parameters can also act individually to affect the outcome. A larger focused-spot radius yields a more uniform laser fluence distribution along a given scan path and increases the number of pulses received at each point, thereby facilitating the formation of continuous and uniform modified regions. However, an excessively large spot compromises spatial resolution and increases the axial depth of focus, which can be expressed as:

(2) $$∆z=\frac{2\lambda n}{{NA}^{2}}$$

The minimum focused-spot radius can be expressed as:

(3) $$\omega_{0}=\frac{0.61\lambda }{NA}$$

represents the wavelength of the laser, n represents the refractive index at the air–SiC interface. According to Equations (2) and (3), when λ and n are fixed, an increase in spot diameter results in a larger axial depth of focus, which contradicts the objective of reducing the modified-layer thickness in this work. Accordingly, an objective with *NA* = 0.42 is selected in this work, yielding a calculated minimum spot diameter of approximately 3 μm. The repetition rate *f* determines the temporal pulse density. In femtosecond-laser–induced nonlinear absorption and avalanche ionization, a sufficiently high pulse density promotes electron accumulation and energy deposition; however, an excessively high frequency can exacerbate heat accumulation, trigger undesired thermal melting, and increase the modified-layer thickness. The scanning speed *v* directly governs overall processing throughput. Moreover, a larger scanning interval leads to discontinuities between adjacent modified tracks, impeding the formation of a complete fracture plane and degrading separation quality, whereas an excessively small interval can induce thermal melting and crack propagation.

In summary, during practical machining, each laser-processing parameter not only affects the outcome directly but also interacts with the others, thereby altering the result. This work focuses on the influence of aberrations caused by the refractive-index mismatch between SiC and air on the processing and does not undertake an in-depth analysis of the parameters themselves. Accordingly, the parameters are set as follows: scanning interval of 10 μm, scanning speed of 100 mm/s, focusing depth of 175 μm (half the wafer thickness), and pulse repetition rate of 100 kHz.

The wafer used in this experiment is a semi-insulating N-doped 4H-SiC wafer produced by Tankeblue Semiconductor. The wafers were cut into 10 × 10 mm samples, with a surface roughness of 3 nm and a thickness of 350 μm. The laser is incident along the [0001] direction [29].

To assess the structural fragility of the femtosecond laser-induced modified layer and its impact on the separation behavior, a mechanical separation experiment was designed and conducted following internal laser scanning to generate the modified region. The laser-processed wafer was bonded between two rigid substrates with ethyl cyanoacrylate, and tensile testing was conducted on an electronic universal testing machine (WDW-20E, Time ShiJin Testing Machine, China) at a constant loading rate. Axial tensile force was gradually applied until fracture or complete separation occurred along the modified layer. Real-time tensile force data were recorded throughout the process, enabling quantitative analysis of lattice damage induced by the femtosecond laser.

In the experiments conducted in this study, five repeated trials were performed under each set of parameters to verify experimental repeatability. For each individual sample, the modified layer thickness and the post-separation surface roughness were measured at five different regions to evaluate their uniformity. Surface microstructures on the sliced face were examined by a scanning electron microscope (SEM, ZEISS SIGMA 300, Germany), and Raman spectra were acquired with a Horiba LabRAM HR 800 (Japan). The macroscopic surface morphology was measured with a Keyence P-030 (Japan) distance-measurement lens (ranging accuracy < 1 μm). During measurement, the lens was fixed while the sliced sample was translated on a two-axis stage to determine the distance from the lens to selected surface points. Within a 10 × 10 mm region of the sliced surface, 500 × 500 points were sampled; the height at each point was recorded, and a best-fit plane was computed and visualized to facilitate an intuitive assessment of the macroscopic topography. Surface roughness was measured by a Mitutoyo Surftest SJ-210 profilometer (Japan).

### 2.2. Aberration Correction Model

**Figure 3:**
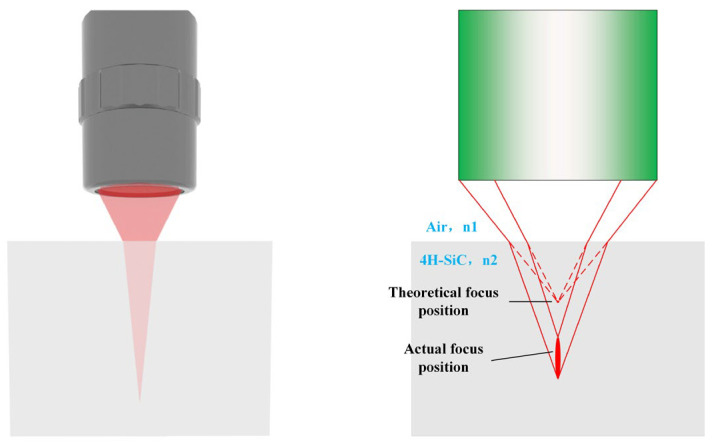
Aberration and focal elongation induced by femtosecond laser focusing in wafer.

The aberration characteristics of the focusing objective used in this study were optimized and compensated during the optical system design stage, ensuring that the residual wavefront distortion has a sub-wavelength-level impact on the focusing performance. The present analysis focuses on the wavefront distortion mechanism induced by the refractive index mismatch at the air–silicon carbide crystal interface. As illustrated in Figure 3, when the collimated femtosecond laser beam is focused by the objective and its ideal geometric focus is focused at a specific subsurface depth within the crystal, the divergence angle at the refractive interface—determined by the beam’s transmission properties—significantly alters the three-dimensional spatial distribution of the focal field. This cross-interface propagation effect results not only in a spatial shift of the actual focal spot but also in asymmetric deformation of the energy density within the focal volume.

Booth suggested that the aberration caused by the refractive index mismatch can be represented by a phase distribution function at the focal point [30]:

(4) $$\varphi \left(\rho ,d\right)=dn_{1}sin\alpha \left(\sum_{n=0}^{\infty }A_{n,0}Z_{n,0}\left(\rho \right)\right)$$

Here, ρ represents the radial distance from the center of the focal point, d is the distance from the focal point to the top surface of the crystal, α is the maximum angle of light incidence incident on the wafer. $NA=n_{1}sin\alpha$, $n_{1}$ and $n_{2}$ represent the refractive indices of air and the silicon carbide. Due to the absence of azimuthal variation, only the *n*th-order, zero-degree Zernike polynomials (with even *n*) are considered here, which can be expressed as:

(5) $$Z_{n,0}\left(\rho \right)=\sqrt{n+1}\sum_{s=0}^{n/2}\frac{{\left(-1\right)}^{s}\left(n-s\right)!}{s!\left(n/2-2\right){!}^{2}}\rho^{n-2s}$$

$A_{n,0}$, $B_{n}\left(\gamma \right)$ can be expressed as:

(6) $$A_{n,0}=B_{n}\left(\alpha \right)-B_{n}\left(\beta \right)$$

(7) $$B_{n}\left(\gamma \right)=\left[1-\frac{n-1}{n+3}{tan}^{4}\left(\frac{\gamma }{2}\right)\right]\frac{{tan}^{n-1}\left(\frac{\gamma }{2}\right)}{2\left(n-1\right)\sqrt{n+1}}$$

Here, $n_{1}sin\alpha =n_{2}sin\beta$.

Table 1 presents the functions of the nth-order, zero-degree Zernike circular polynomials and their corresponding types of optical aberrations. As shown in Table 1, the aberrations induced by the refractive index mismatch between air and the silicon carbide crystal in the experiment are primarily low-order spherical aberrations.

To effectively mitigate the adverse effects of low-order spherical aberration caused by refractive index mismatch on laser focusing performance, and to enhance both the focal spot quality and the uniformity of energy density distribution, an aspheric lens was designed and introduced in this study as a pre-correction optical element based on the system wavefront aberration theory. The surface profile of the aspheric lens can be expressed as:

(8) $$z=\frac{cr^{2}}{1+\sqrt{1-\left(1+k\right)c^{2}r^{2}}}+a_{4}r^{4}+a_{6}r^{6}+a_{8}r^{8}+\cdots$$

Each term with coefficients such as *a*_4_, *a*_6_… in the formula represents the deformation of the aspheric surface relative to the base conic surface. In the formula, *r* is the radial distance from a point on the aspheric surface to the optical axis. The specific values of these coefficients were optimized by Zemax (Zemax OpticStudio 2024 R1).

## 3. Results

### 3.1. Analysis of Aberration Calculation

**Figure 4:**
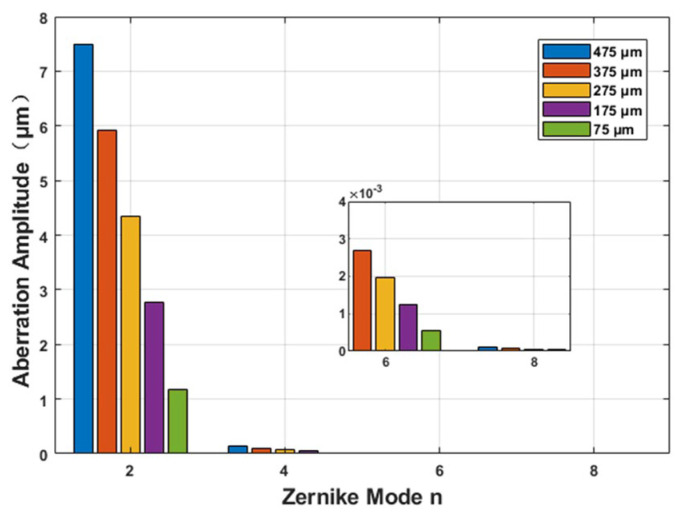
Zernike coefficients of the nth-order, zero-degree terms at different depths.

To analyze the impact of aberration on the processing performance, the aberrations under different parameter conditions were calculated based on Equation (5), and the results are shown in Figure 4. At the same focusing depth, the aberration amplitude reaches its maximum at *n* = 2 (defocus term), while the magnitude of the Zernike coefficients exhibits an exponential decay trend with increasing n. Notably, when *n* ≥ 4, the coefficients decrease significantly to a negligible level. This observation indicates that the aberrations induced by refractive index mismatch are primarily concentrated in the low-order terms, with second-order spherical aberration and defocus being the most prominent, whereas higher-order even terms contribute minimally to the overall aberration distribution.

Further analysis of the influence of focusing depth reveals a clear trend. As the focal point shifts deeper into the sample, the amplitude of the second-order aberration terms increases significantly. This behavior indicates that the system is more sensitive to low-order spherical aberrations with increasing focusing depth. This behavior can be attributed to the fact that, during beam propagation across the interface between different media, the wavefront distortion induced by the refractive index gradient exhibits a stronger response in low-order modes. Therefore, when correcting and compensating for systematic aberrations caused by refractive index mismatch, optimization should focus primarily on the low-order Zernike coefficients, particularly the correction of secondary spherical aberration. In contrast, the influence of higher-order even aberrations (above the fourth order) can be neglected within the error tolerance range, thereby reducing the dimensionality and computational complexity of the correction process and enhancing both its efficiency and robustness.

### 3.2. Effect of Aberration on the Focal Aspect Ratio

As demonstrated by the computational results in Section 3.1, aberrations caused by the refractive index mismatch become more pronounced with increasing focusing depth. To simplify the design of the aberration correction system and accommodate the practical limitations associated with the separation process, the focusing depth during actual processing was set to 175 μm below the crystal’s top surface. This was achieved by programming the five-axis motion stage to translate along the Z-axis, ensuring precise focal positioning.

**Figure 5:**
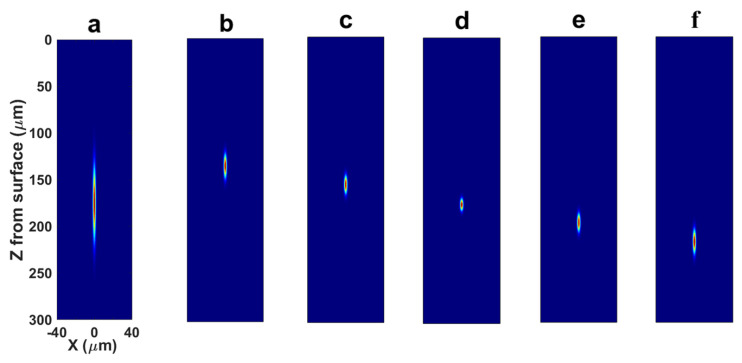
Comparison of focusing performance before and after aberration correction: (**a**) Focal spot at a depth of 175 μm without aberration correction. (**b**–**f**) Focal spots with aberration correction at different depths: (**b**) 135 μm; (**c**) 155 μm; (**d**) 175 μm; (**e**) 195 μm; (**f**) 215 μm.

The stage executed uniform scanning at a constant velocity to complete the full-wafer laser processing. To compensate for the aberrations induced by refractive index mismatch at this specific subsurface depth, an aspheric lens was specifically designed and integrated into the optical system to pre-compensate wavefront distortions. As illustrated in Figure 5, a simulation analysis was conducted to compare the focusing performance of the laser spot with and without the aberration-correction system. In addition, the focusing behavior at different depths was simulated to evaluate the performance of the correction system under various focusing conditions.

Without aberration correction, the abrupt change in refractive index at the air–crystal interface distorts the initially spherical wavefront during focusing, resulting in focal elongation along the optical axis. The elongation causes a reduction in laser fluence at the focal point due to the expansion of the focal volume. As a result, the laser fluence within a unit volume may fall below the threshold required for nonlinear absorption or optical breakdown in the lattice, leading to insufficient lattice damage. This, in turn, affects the post-separation surface quality and may cause discontinuities at the separation interface. By comparing Figure 5b–f, it can be observed that the correction performance of the system deteriorates at depths other than 175 μm. This occurs because variations in the focusing depth induce axial focal shifts and changes in the effective pupil filling, leading to a redistribution of the equivalent NA and aberration weights. As a result, the original phase mask no longer corresponds to the optimal energy convergence, causing axial energy broadening and an increased aspect ratio of the focal spot.

**Figure 6:**
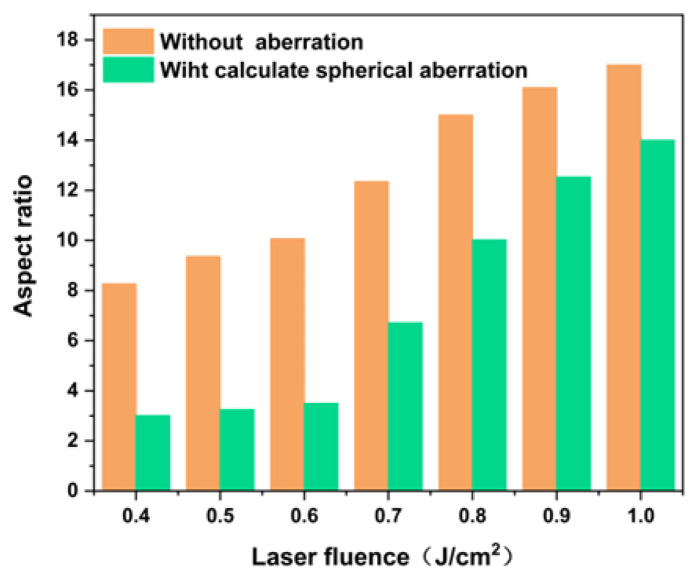
Focal aspect ratios before and after aberration correction under different laser fluences.

As shown in Figure 6, the aspect ratio of the focal spot under different laser fluence conditions is compared before and after aberration correction. For a given objective lens, this value directly reflects the modified layer thickness. The results indicate that without aberration correction, the aspect ratio ranges from 8.27 to 17. As the laser fluence increases, the aspect ratio of the focal spot gradually rises. Under high laser fluence conditions, the optical Kerr effect causes a local increase in refractive index within the crystal [31], where the refractive index at the center of the beam becomes higher than at the periphery, acting like a gradient-index lens and leading to further lateral compression of the beam. Meanwhile, the combined effects of nonlinear phase distortions and plasma-induced phenomena lead to a pronounced elongation of the focal depth, resulting in an increased aspect ratio of the focused spot as the laser fluence rises. With the incorporation of the aberration correction module, the aspect ratio is effectively maintained below 4 under low-fluence conditions. As the laser fluence continues to rise, although the aberration correction still provides partial improvement compared to the uncorrected condition, its overall suppression effect becomes less pronounced. This is attributed to the strong dynamic nature of the self-focusing effect induced by nonlinear refractive index changes, causing the aberrations to vary rapidly in both spatial and temporal domains, which cannot be fully compensated by a static correction module. Therefore, selecting an appropriate energy density is crucial for controlling the thickness of the modified layer in practical processing.

### 3.3. Effect of Aberrations on Separation Surface

**Figure 7:**
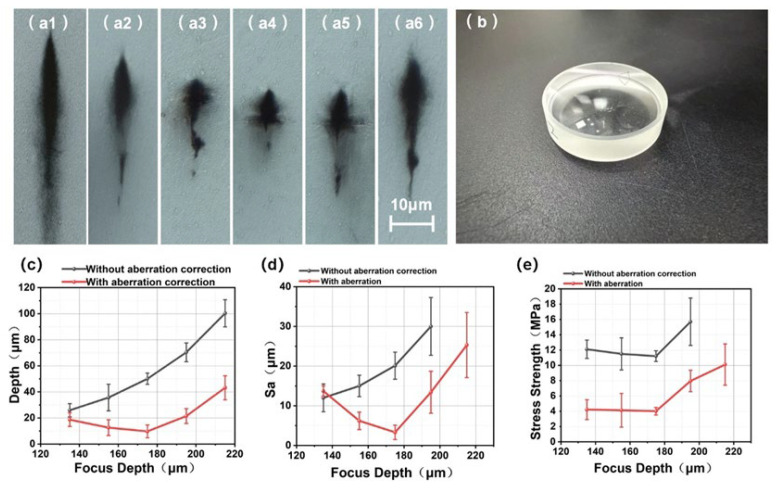
Slicing performance of the correction lens at different depths. (**a1**) Without the correction lens; (**a2**–**a6**) With the correction lens, the focusing depth: (**a2**) 135 μm; (**a3**) 155 μm; (**a4**) 175 μm; (**a5**) 195 μm; (**a6**) 215 μm; (**b**) The correction lens. (**c**) Modified-layer thickness. (**d**) Surface roughness. (**e**) Tensile stress.

To evaluate the correction performance of the lens at different focusing depths, a series of experiments was conducted using the aspheric lens configuration shown in Figure 7b. The aspheric lens is fabricated from fused silica to ensure high transmittance in the infrared range and a high laser damage threshold. Its effective clear aperture is 2 inches, meeting the beam size requirements of the femtosecond laser focusing system. Figure 7(a1–a6) presents the modified layer morphologies at various depths with and without the correction lens, while Figure 7c–e respectively summarize the damage-layer thickness, surface roughness, and tensile stress obtained under different processing parameters. The results reveal that, without the correction lens, both the modified layer thickness and the surface roughness of the sliced surface increase rapidly with focusing depth. As the focal depth increases, the optical path within the crystal becomes longer, and the wavefront distortion induced by refractive-index mismatch becomes more severe. Consequently, focal shifts and spot broadening occur along the optical axis, leading to nonuniform energy deposition and a thicker modified layer. In addition, deep focusing may trigger dynamic nonlinear effects such as self-focusing and plasma scattering. The competition among these effects varies with depth, resulting in greater focal instability and further degradation of energy uniformity. With the correction lens, the modified-layer thickness, the tensile stress required for slicing, and the surface roughness at the design depth all decrease significantly. As the focusing depth deviates from the design value, these parameters gradually deteriorate. Inspection of Figure 7(a1–a6) shows that when the focusing depth deviates from the design depth, the damage region enlarges significantly and its edges become progressively blurred. At the design depth, the laser energy is more tightly confined, leading to more complete lattice modification within the crystal, which is consistent with the minimum tensile stress required for slicing at this depth. It is further observed that negative deviation (shallower than the design depth) leads to much slower degradation in both modified-layer thickness and surface quality compared with positive deviation (deeper than the design depth). This occurs because, under negative deviation, the induced aberration is smaller than the static compensation provided by the lens (slight overcompensation), allowing the focusing performance to remain relatively stable. As a result, focal distortion is minor, energy confinement changes little, and the degradation of slicing quality is slow. In contrast, when the focusing depth deviates positively, the aberration induced by refractive-index mismatch exceeds the static compensation capability of the lens (insufficient compensation), causing a forward focal shift and severe axial elongation or even splitting of the focal spot, leading to a double-peaked trailing intensity profile. Consequently, the local peak intensity decreases while the energy spreads axially, resulting in rapid deterioration of the slicing performance. Nevertheless, compared with the uncorrected case at the same depths, a certain degree of improvement is still achieved. Moreover, the experiments reveal that without the correction lens, successful slicing cannot be achieved when the focusing depth exceeds 215 μm. This is likely because the large aberration at such depths causes severe focal degradation, preventing effective modification inside the crystal and thus leading to slicing failure.

**Figure 8:**
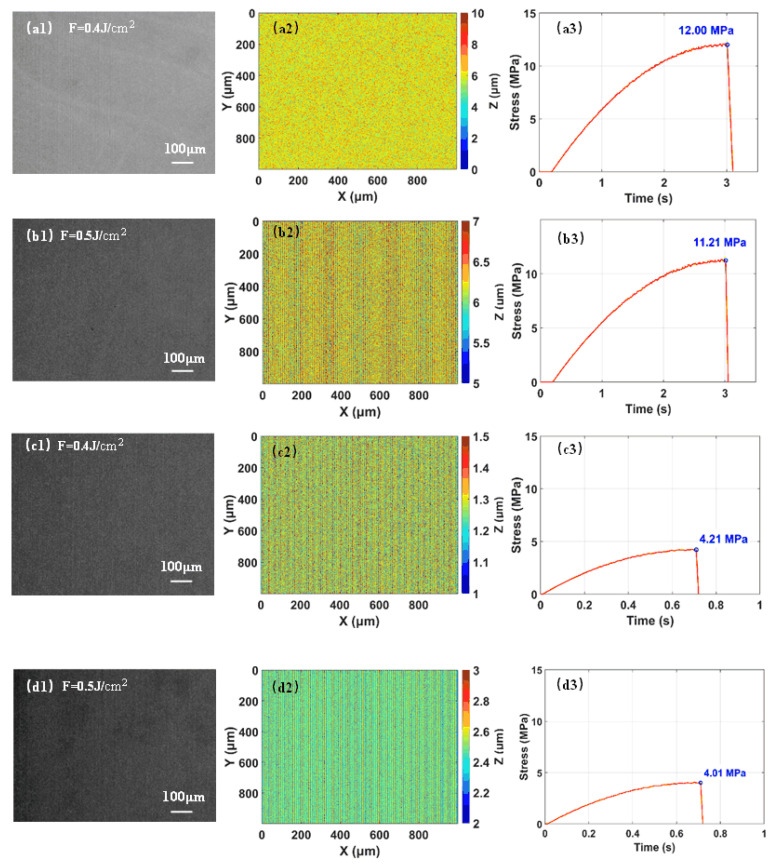
Processing results at different laser fluences with and without aberration correction ((**a**,**b**): without correction, (**c**,**d**): with correction). (**1**) Separation surface morphology; (**2**) Surface profile pseudo-color maps; (**3**) Tensile stress curves from mechanical separation tests.

The surface morphology and mechanical separation characteristics of the samples were comparatively analyzed under conditions with and without aberration correction, as shown in Figure 8. Without aberration correction, when the laser fluence was set to 0.4 J/cm^2^, the maximum tensile stress required for wafer separation reached 12.00 MPa. As the fluence increased to 0.5 J/cm^2^, the maximum tensile stress decreased to 11.21 MPa. This phenomenon is primarily attributed to the significantly enhanced localized energy deposition of the femtosecond laser within the crystal at higher fluence levels, which intensifies nonlinear absorption and multiphoton ionization effects. As a result, the density of micro-defects, microcracks, and voids within the modified layer increases, substantially weakening its structural integrity and mechanical strength. After the integration of an optical correction system into the beam path, wavefront aberrations were effectively compensated using an aspheric correction lens. Consequently, the tensile stress required for separation decreased to 4.21 MPa at a fluence of 0.4 J/cm^2^, indicating a significant improvement in mechanical separation performance. Under identical processing parameters, the maximum tensile stress required for separation was significantly lower than that without aberration correction. As observed in Figure 8(a1–d1), the separation surface without aberration correction lacks distinct longitudinal scanning stripes, and the overall surface roughness is relatively high. This is mainly because spherical aberration distorts the energy distribution of the focused spot from an ideal Gaussian profile, resulting in uneven formation of the laser-modified layer and reduced surface quality after separation. This indicates that aberration correction effectively improved the laser focusing performance, particularly along the optical axis, and notably suppressed the increase in modified layer thickness caused by focal spot elongation. By enhancing the peak laser fluence in the interaction region and improving laser energy utilization, the correction promoted more uniform formation of the modified layer, thereby reducing the mechanical strength of the separation interface and enhancing the stability and reliability of the slicing process.

**Figure 9:**
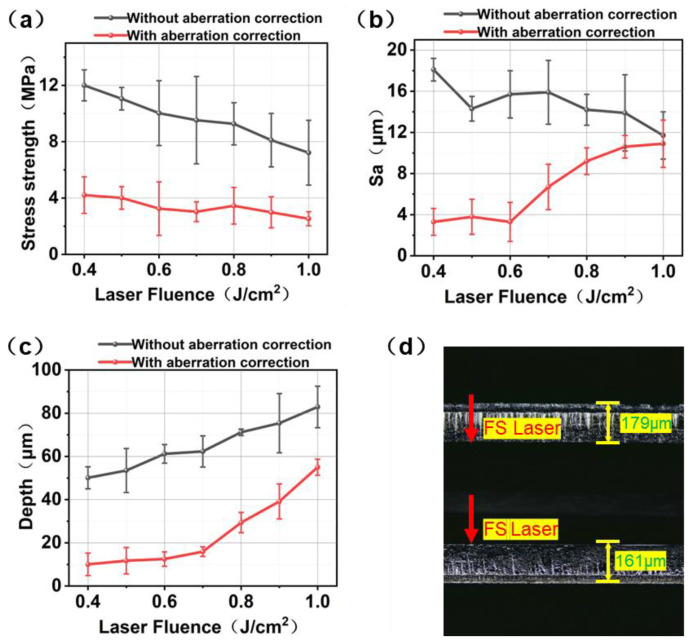
(**a**) Tensile stress curves. (**b**) Surface roughness curves. (**c**) Damage layer thickness curves. (**d**) Cross-sectional view of the sliced wafer (Laser fluence of 0.6 J/cm^2^, focusing depth of 175 μm, with the correction lens).

After the separation experiments, a surface profilometer was used to measure the surface roughness, serving as an indicator of the post-separation surface quality. In this study, we recorded the maximum tensile stress required for separation, the resulting surface roughness, and the modified layer thickness for wafers processed at different laser fluences, both with and without aberration correction. The results are summarized in Figure 9.

Without aberration correction, the maximum tensile stress required during separation gradually decreases with increasing laser fluence. This trend is primarily attributed to the enhanced effective energy deposited in the focal region at higher fluence, which intensifies the localized damage to the crystal lattice and consequently reduces the external load required for separation. However, it is worth noting that as the laser fluence continues to increase, the spatial extent of the damage region also expands, resulting in a significant increase in the axial thickness of the modified layer. The surface roughness Sa after separation ranges from 18.1 μm to 11.8 μm, indicating that while scanning laser fluence has some influence on improving surface quality, the effect remains limited.

After introducing the aberration correction system, the tensile stress required for separation is markedly reduced compared to the uncorrected condition. This indicates that aberration compensation effectively optimizes the focal spot shape and energy deposition profile, thereby enhancing the efficiency of modified layer formation. However, when the laser fluence is further increased to 0.7 J/cm^2^, experimental results reveal a sharp rise in both the modified layer thickness and the surface roughness of the separated interface. This is primarily due to the strong nonlinear refractive response induced within the crystal at excessively high fluence, which renders the static wavefront-based aberration correction ineffective. Particularly in terms of surface roughness, when the laser fluence increases to 1.0 J/cm^2^, the Sa value rises to a level comparable to that without aberration correction, indicating that excessive laser fluence diminishes the effectiveness of the aberration correction. As a result, the focal spot aspect ratio becomes uncontrollable, and the spatial distribution of laser energy within the material becomes non-uniform. The degradation of focusing performance ultimately leads to excessive modified layer thickness and deteriorated interface roughness, thereby compromising the separation quality and process stability.

Increasing the laser fluence enhances the localized damage to the crystal and reduces the tensile stress required for separation, but also leads to a thicker damage layer. After introducing the aberration correction system, the laser focusing performance is significantly improved, resulting in a notable reduction in the tensile stress required for separation. When the laser fluence exceeds 0.6 J/cm^2^, the refractive index variation in the SiC crystal caused by high energy density gradually undermines the effectiveness of the static aberration correction system, leading to a sharp increase in both the damage layer thickness and the surface roughness after separation. Therefore, it can be concluded that the static aberration correction system performs well when the fluence is ≤0.6 J/cm^2^. At a fluence of 0.6 J/cm^2^, the best separation results were obtained, with a tensile stress of 4.01 MPa, a modified layer thickness of 9.72 μm, and a post-separation surface roughness (Sa) of 3.3 μm.

In summary, the static correction system effectively improves slicing performance near the design depth, but this benefit diminishes as the depth deviates from the design value. Experiments further show a markedly higher tolerance to negative deviation (shallower focus) than to positive deviation, indicating better robustness to slightly shallower focusing in practical applications.

### 3.4. SEM and Raman Spectroscopy Analysis

**Figure 10:**
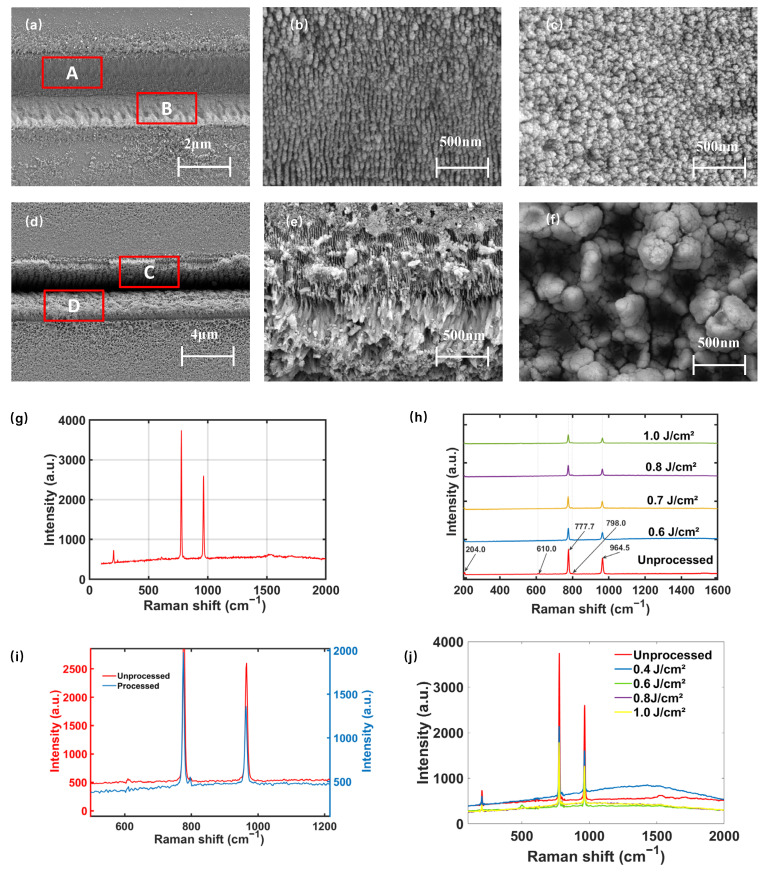
SEM image of (**a**) Scanning laser fluence of 0.6 J/cm^2^; (**b**) Region A; (**c**) Region B; (**d**) Laser fluence of 1.0 J/cm^2^; (**e**) Region C; (**f**) Region D; Raman spectra of: (**g**) Unprocessed sample; (**h**) Samples under different processing conditions; (**i**) Processed and unprocessed samples (partial); (**j**) Samples under different processing conditions (in the same coordinate system).

To optimize the processing parameters, the separation surface morphology at different laser fluences with aberration correction was systematically characterized. Samples were examined by a field-emission scanning electron microscope to analyze the microstructural features of the separation interface and their formation mechanisms. Figure 10a–f present the surface micro-morphologies at different scales after slicing under various laser fluences. When the laser fluence is 0.6 J/cm^2^, laser scanning produces uniformly distributed but shallow microgrooves. When the fluence increased to 1.0 J/cm^2^, distinct recast layers appeared on both sides of the ablation lines and expanded outward, indicating that the focal temperature exceeded the evaporation threshold of SiC. The formation of recast layers disrupted groove uniformity, reduced modified layer consistency, and degraded the flatness and roughness of the separation surface. As shown in Figure 10f, further increases in fluence significantly enlarged the heat-affected zone (HAZ), and the regions adjacent to the ablation lines developed numerous pits due to localized heating, intensifying surface non-uniformity.

Raman spectrum with an excitation wavelength of 532 nm was employed to characterize samples processed under different conditions, aiming to analyze the structural evolution, carrier concentration variations, and residual stress changes in the wafer surface before and after separation. As shown in Figure 10g, the Raman spectrum of the unprocessed sample exhibits five characteristic first-order intrinsic modes of 4H-SiC: E_2_(TA) ≈ 204.0 cm^−1^, A_1_(LA) ≈ 610.0 cm^−1^, E_2_(TO) ≈ 777.7 cm^−1^, E_1_(TO) ≈ 798.0 cm^−1^, and A_1_(LO) ≈ 964.5 cm^−1^. After processing, distinct SiC peaks remain visible at 777.7 cm^−1^ and 964.5 cm^−1^, indicating that the crystalline phase and fundamental lattice framework were preserved. As illustrated in Figure 10i, compared with the unprocessed sample, the Raman peak intensities of the separated wafer surface decreased significantly, while the full width at half maximum (FWHM) remained essentially unchanged. This behavior suggests that the reduction in peak intensity is mainly attributed to extrinsic signal attenuation rather than intrinsic lattice degradation. In this study, the attenuation primarily originated from increased surface roughness after separation, which reduced specular reflection and enhanced scattering, as well as possible input–output optical losses induced by surface oxides. The unchanged FWHM indicates a negligible average thermal effect during processing, further confirming the “cold processing” nature of femtosecond laser irradiation. As shown in Figure 10j, some samples exhibited a weak band near 1500 cm^−1^ after processing, which may be associated with the formation of amorphous carbon. In addition, no discernible systematic Raman shifts in the main peaks were observed, demonstrating that the residual average stress in the samples remained limited. Although internal stresses are inevitably introduced during femtosecond laser scanning of SiC wafers, the Raman spectra of the separated surfaces confirm that such stresses were effectively released during the subsequent mechanical separation using a universal tensile testing machine.

## 4. Discussion

In this study, the feasibility and processing performance of the static aberration correction system have been validated through a series of experiments. Here, we further compare the processing results obtained in this work with those reported for active optical aberration correction systems. The comparison results are summarized in the Table 2 below.

As shown in Table 2, it can be observed that the proposed method significantly increases the scanning speed while effectively controlling the thickness of the modified layer. At the same time, compared with existing approaches, this work enables the fabrication of thinner wafers while maintaining better post-exfoliation surface roughness.

Compared with active optical aberration correction systems, the aspheric lens designed in this study reduces the cost of the aberration correction module by approximately 90%. In addition, active correction methods typically require wavefront sensing, phase calibration, and closed-loop optimization, relying on real-time control algorithms for dynamic compensation. In contrast, the static optical correction approach proposed here does not require additional wavefront measurement or real-time adjustment once the system is established. This reduces system alignment and calibration complexity while improving overall system stability.

Although the proposed static aberration correction method demonstrates clear advantages in processing performance, there is still room for further improvement. For instance, different material synthesis routes may affect the microstructure and defect distribution, thereby influencing parameters such as laser absorption and transmission. Moreover, processing parameters including scanning speed, number of passes, and laser fluence have direct impacts on both processing quality and efficiency, and require further optimization. In addition, experimental observations indicate that variations in wafer fabrication methods can lead to differences in microstructural uniformity, which, due to the underlying mechanisms of femtosecond laser–material interaction, may result in variability in processing outcomes under identical conditions. To enhance the robustness and general applicability of the process, further refinement and optimization are still needed.

## 5. Conclusions

In this paper, a static aberration correction method was proposed for femtosecond laser-based slicing of 4H-SiC wafers. Simulation analysis revealed that the aberrations caused by the refractive index mismatch between air and SiC are predominantly composed of low-order spherical aberrations. Based on this finding, a static aberration correction system employing an aspheric lens was designed and implemented. Using this system, successful separation of a 10 mm × 10 mm × 161 μm wafer was achieved. Experimental results demonstrated that the performance of the correction system is strongly influenced by laser fluence, with optimal processing results obtained at a fluence of 0.6 J/cm^2^. Under this condition, the modified layer thickness was reduced to 9.72 μm, the maximum tensile stress required for separation was 4.01 MPa, and the surface roughness reached Sa = 3.3 μm. The aberration correction system greatly improves the focusing quality of the laser spot and reduces the thickness of the damage layer, leading to improved uniformity of the modified region. As a result, the wafer yield is enhanced, making it easier to achieve the production of large-area, ultrathin wafers. In summary, although the proposed correction method imposes certain limitations on the selection of laser parameters, it greatly reduces system complexity and improves operational stability under long-term, high-intensity conditions in fixed-process workflows. As a result, this technique holds promise for the mass production of large-area, ultra-thin wafers such as 6-inch or 8-inch substrates.

## Figures and Tables

**Table 1:** Functions of nth-order, zero-degree Zernike circular polynomials and their corresponding types of optical aberrations.

*n*	$Z_{n,0}\left(\rho \right)$	Description
2	$\sqrt{3}\left(2\rho^{2}-1\right)$	Defocus
4	$\sqrt{5}\left(6\rho^{4}-6\rho^{2}+1\right)$	Primary spherical aberration
6	$\sqrt{7}\left(20\rho^{6}-30\rho^{4}+12\rho^{2}-1\right)$	Secondary spherical aberration
8	$\sqrt{9}\left(70\rho^{8}-140\rho^{6}+90\rho^{4}-20\rho^{2}+1\right)$	Tertiary spherical aberration

**Table 2:** Comparison of processing results between different studies and the present work.

Ref	Material	λ (nm)	Pulse Duration (fs)	Roughness (µm)	Focus Depth (µm)	Scan Speed (mm/s)	Modified Layer Thickness (µm)
Ref. [18]	4H-SiC	1064	220	1.8	250	0.1	2
Ref. [10]	4H-SiC	780	220–6000	5	250	0.1	24
Ref. [15]	4H-SiC	1030	290–15,000	5.3–6.53	250	2	<100
This Work	4H-SiC	1030	256	3.2	161	100	9.72

## Data Availability

The original contributions presented in the study are included in the article. Further inquiries can be directed to the corresponding author.
